# *Caenorhabditis elegans* *ddx-15* helicase fails to complement loss of Prp43p in *Saccharomyces cerevisiae*

**DOI:** 10.17912/micropub.biology.001113

**Published:** 2024-01-22

**Authors:** Jonathan E Karpel

**Affiliations:** 1 Biology, Southern Utah University, Cedar City, Utah, United States

## Abstract

Helicase proteins have important roles in many aspects of RNA metabolism in the cell. The function of these highly conserved proteins is commonly preserved between organisms, yet in a few cases these homologues are found to have slightly different biochemical functions. Prp43 is a protein with varied roles in yeast, but here we show that the *C. elegans* homologue of this protein is unable to rescue the loss of Prp43p. By employing a transcriptional repression experiment, the expression of DDX-15 protein in yeast is not enough to complement the loss of Prp43p, which is a yeast essential protein.

**Figure 1. C. elegans DDX-15 protein fails to complement the loss of yeast Prp43p in vivo:**
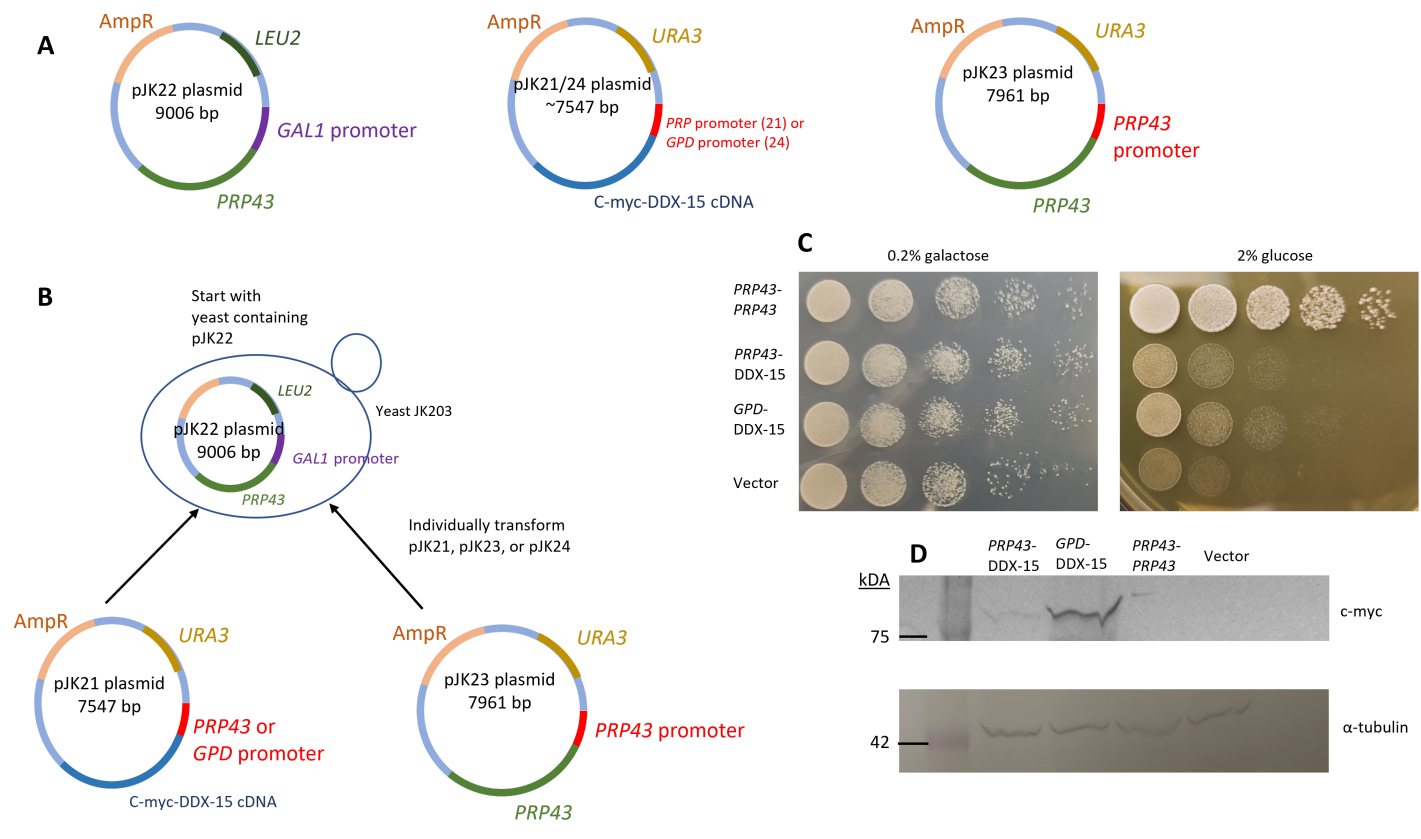
**A** , Plasmids were constructed to place the *PRP43* gene under the control of either *GAL1* or *PRP43* promoters, and the DDX-15 cDNA (N-terminal c-myc tag) under the control of either *PRP43* or *TDH3* promoter (GPD). **B** , Yeast was transformed with pJK22 and either pJK21, pJK23 or pJK24 to set up a transcriptional repression experiment. Yeast strains were maintained at 30 ^O^ C on minimal media containing 0.2% galactose, 1.8% raffinose plates lacking uracil and leucine. **C,** Strains were grown in minimal media containing 0.2% galactose, 1.8% raffinose to saturation at 30 ^O^ C , and serial dilutions were spotted onto plates with 0.2% galactose, 1.8% raffinose or with 2% glucose and grown at 30 ^O^ C. Growth was observed after 2-3 days. **D** , Strains were grown in minimal media containing 0.2% galactose, 1.8% raffinose to saturation at 30 ^O^ C, total protein was isolated and blotted to PVDF. Membranes were probed with either c-myc or anti-tubulin antibody and visualized with a colorimetric kit.

## Description

RNA binding proteins have been shown to be involved with numerous important processes within the cell (Gerstberger et al. *,* 2014). One critical function of RNA binding proteins is transcriptional regulation, especially concerning how pre-mRNAs are spliced during gene expression. Malfunctions of splicing patterns have been linked to a plethora of diseases; therefore it is important to understand how RNA-binding proteins act to regulate the splicing process (Gebauer et al. *,* 2021).

The pre-mRNA splicing process (and the proteins involved) in the yeast *Saccharomyces cerevisiae* has been elucidated quite well, but the same cannot be said for the nematode *Caenorhabditis elegans.* There are representative *C. elegans* orthologs for each yeast splicing protein, but it is tricky to assume that orthologous proteins would have the same function between species. Interestingly, several of the putative spliceosomal genes in *C. elegans* (specifically the *mog* genes, *prp-17* , and *prp-19* ) (Belfiore et al., 2004; Kerins et al., 2010; Gutnik et al., 2018) display overt phenotypes in germ cell maturation, suggesting a role for splicing in regulating cell fate decisions such as entry into meiosis. For example, mutations/RNAi of the *mog* genes result in a “masculinization of the germline” (mog) phenotype, suggesting these genes influence the switch from spermatogenesis to oogenesis (Kerins et al., 2010; Gutnik et al., 2018) .

The exact mechanism(s) for how these pre-mRNA splicing factors function to affect germ cell fate versus meiotic development is not entirely clear. Many of these factors seem to act redundantly, and it may just be a general requirement of splicing, or correct structure of the exon junction complex, for example, that is needed for proper expression of genes in the canonical germline development pathways.

Another presumed member of the pre-mRNA splicing pathway in *C. elegans* is DDX-15 helicase. This protein is orthologous (87% identity) to yeast Prp43p, which is responsible for one of the final steps in splicing (Arenas and Abelson, 1997) , yet to date there is no evidence that this protein functions as part of the spliceosome in *C. elegans* . *ddx-15* was not identified in the screens that led to the identification of the *mog* genes ( *mog* -1, 4 and 5 are splicing factor homologues, see Puoti and Kimble, 1999 and 2000), nor was it found in a separate proteomic analysis of spliceosomal components (Doherty et al., 2014) . Yet *ddx-15* mutants display a germline defect similar to the phenotypes exhibited by mutations in the *mog* genes.

In *S. cerevisiae* , there is evidence of multiple biochemical functions for Prp43 protein that are independent of the protein’s role as part of the spliceosome, for example in ribosome biogenesis (Leeds et al., 2006) . In *C. elegans,* there is one study that suggests a role in repressing RNA editing and suggested that DDX-15 does in fact interact with other spliceosomal components, but not MOG-1, 4 or 5 (Tariq et al., 2012) . Considering all these separate studies, it is possible the *C. elegans* DDX-15 protein has roles in RNA metabolism other than simply being a part of the spliceosome.

We decided to test the ability of DDX-15 to rescue the function of Prp43p in *S. cerevisiae* , utilizing a plasmid shuffle-like experiment. *PRP43* is an essential gene in yeast and mutants are inviable, so we decided to place the *PRP43* gene under a galactose-inducible, glucose-repressible promoter ( *GAL1* ) in *S. cerevisiae* , and then test the ability of the DDX-15 protein to rescue the transcriptional repression of Prp43p *in vivo* . We designed four plasmids that would enable us to control the expression of the *ddx-15* and *PRP43* genes individually (Table 1).

We first transformed a plasmid containing the wild-type *PRP43* gene under the control of the inducible *GAL1* promoter (pJK22; Figure 1A ) into yeast strain BY4743, and sporulated this strain to produce the double *prp43-Δ/prp43-Δ* mutant. This resulted in yeast strain JK203 that could be maintained on a minimal media containing 0.2% galactose, 1.8% raffinose and lacking leucine to stabilize the plasmid.

Into the JK203 strain we individually transformed plasmids and isolated yeast strains containing the following genetic constructs: yeast containing pJK21 with the *C. elegans* *ddx-15* cDNA under control of the native *PRP43* promoter (JK207), or the *TDH3* overexpression promoter (JK208), and yeast JK205 containing pJK23 with the wild type *PRP43* under the native *PRP43* promoter ( Figure 1A ). We also transformed the empty vector (pRS416) into strain JK203 as a control. The transformation overview is given in Figure 1B .

All these double-transformed strains were tested for growth on media containing 0.2% galactose and 1.8% raffinose and lacking both leucine and uracil (left panel, Figure 1C ). We isolated total protein from all yeast strains and used western blotting to confirm protein expression. We were not able to assess the expression of yeast Prp43p as we lacked a proper antibody, but we can assume the Prp43 protein is expressed from plasmids pJK22 and pJK23 since expression is essential for growth. We tested the anti-DDX15 antibody (Santa Cruz; sc-271686) but it failed to bind to either Prp43 or DDX-15 proteins. Therefore, we included an N-terminal c-myc tag on the *ddx-15* cDNA in pJK21 and pJK24 and were able to confirm expression of DDX-15 protein ( Figure 1D ).

We then tested whether expression of the DDX-15 protein could rescue the loss of Prp43p by spot testing the same strains on media containing 2% glucose to repress the expression of Prp43p from plasmid pJK22. Expression from the *GAL1* promoter is strongly repressed in the presence of glucose (Flick and Johnston, 1990) .

Strains transformed with the native *PRP43* gene under control of the native promoter (pJK23) grew much better on 2% glucose plates compared to strains transformed with the *ddx-15* cDNA (right panel, Figure 1C ). Overexpression of the DDX-15 protein did not significantly improve the growth of the yeast. This suggests that the DDX-15 protein does not function well enough to rescue the loss of Prp43p in yeast.

There are several reasons why the DDX-15 protein apparently is not able to rescue the loss of the Prp43 protein. Protein folding and/or post-translational modifications may be a factor as there may not be the unique chaperone proteins available to correctly assist the proper folding of the protein. Prp43p may also have some biochemical functions specifically in yeast that are not properly catalyzed by the DDX-15 protein. Also, there may be binding partners specific to DDX-15 required for proper function that are lacking in *S. cerevisiae.* More research will entail identification of binding partners for DDX-15 that will help us to elucidate how this protein is functioning in *C. elegans* .

## Methods

**Strains and antibodies.** *S. cerevisiae* strains used in this study are listed in Table 2. Dropout media (Takara Bio) contained YNB (+ammonium sulfate) w/o amino acids supplemented with complete supplement mixture lacking leucine, uracil, or both. Antibodies that were commercially purchased are as follows: DDX-15 (sc-271686, Santa Cruz Biotech), alpha tubulin (sc-53030, Santa Cruz), m-IgG Fc BP-HRP secondary antibody (sc-525409, Santa Cruz), and Myc antibody (sc-551062, Santa Cruz).

**Plasmids used in this study.** Plasmids used in this study are listed in Table 1. Plasmids were constructed using Gateway cloning (ThermoFisher) and verified through sequencing. Plasmids pJK21 and pJK24 containing the *C. elegans* *ddx-15* cDNA were modified with a N-terminal c-myc tag sequence. Plasmids pRS415 and pRS416 were a gift from L. Bisson.

**Spot testing yeast strains.** Yeast cells were grown to log phase overnight in YNB with 0.2% galactose, 1.8% raffinose at 30 deg. C. Cells were then diluted through 10-fold serial dilutions and spotted on YNB plates with either 0.2% galactose and 1.8% raffinose, or 2% glucose. Plates were incubated at 30 deg. C until colonies appeared.

**Yeast complementation assay** . The ability for *C. elegans* *DDX-15* protein to rescue Prp43p function in *Saccharomyces cerevisiae* was investigated using a transcriptional repression experiment. *S. cerevisiae* *prp43-Δ/+* strain BY4743 was transformed with plasmid pJK22 ( *GAL1* : *PRP43* , *LEU2* , Figure 1B ) using standard procedures. The transformed strain was sporulated in 2% KOAC, spores were separated and grown on YNB, 0.2% galactose, 1.8% raffinose plates lacking leucine, and homozygous *prp43-Δ* colonies that grew with pJK22 were identified through colony PCR using deletion knockout primers (sequences available upon request). Strain BY4743 was used in the systematic deletion project (Winzeler et al., 1999) and is *MAT* a/α. The *prp43-Δ/+* strain was only available as a homothallic diploid from the deletion collection. When homothallic strains are sporulated cells enter meiosis and you get haploid cells that are either *PRP43* or *prp43-Δ* . Spores can be separated and then a single spore can switch mating types while forming a colony, and mate with cells in the same colony that have not switched. The resulting colony then is predominantly diploid. Since colony growth is dependent on successful segregation of the pJK22 plasmid into each spore, this could be used to screen colonies for the homozygous *prp43-Δ* mutation.

*S. cerevisiae* *prp43-Δ/Δ* (pJK22) strains were transformed with plasmids containing either wild-type yeast *PRP43* under native promoter (pJK23), *C. elegans ddx-15* with native *PRP43* promoter (pJK21) or *TDH3* overexpression promoter (pJK24), or empty vector (PRS416). Positive transformants were maintained on YNB, 0.2% galactose, 1.8% raffinose plates lacking leucine and uracil. Transformed strains were spot tested by plating serial dilutions on YNB plates with 2% glucose, lacking leucine and uracil. Control plates containing 0.2 % galactose were also spot tested to ensure that the same amount of yeast was plated across all samples.

**Western blotting.** Yeast strains were grown to saturation in YNB with 0.2% galactose and 1.8% raffinose with shaking at 30 deg. C. Total protein was isolated using a modified alkaline lysis procedure (von der Haar, 2007) . Briefly, cell pellets were treated with sodium hydroxide, SDS, and beta-mercaptoethanol, boiled, and then neutralized with acetic acid. Cell extract was separated by SDS-PAGE using a 7.5% gel and transferred to PVDF. The blots were probed with primary antibodies followed by secondary goat anti-mouse antibodies conjugated with HRP. Membranes were washed with TBS-tween and visualized with the Opti-4CN colorimetric kit (Bio-Rad).

## Reagents

Table 1. Plasmids used in this study

**Table:** 

**Plasmid**	**Coding region**	**Promoter**	**Vector/Marker**	**Source**
pJK21	*S. cere* *PRP43*	*PRP43*	pRS416/URA3	This study
pJK22	*S. cere* *PRP43*	*GAL1*	pRS415/LEU2	This study
pJK23	*C. elegans DDX-15* (N-term c-myc)	*PRP43*	pRS416/URA3	This study
pJK24	*C. elegans DDX-15* (N-term c-myc)	*TDH3(GPD)*	pRS416/URA3	This study
pRS416	Empty	Empty	URA3	Sikorski, R. and P. Hieter (1989)

Table 2. Yeast strains used in this study

**Table:** 

**Strain**	**Genotype**	**Source**
BY4743	*MATa/MATalpha his3Δ/his3Δ leu2Δ/leu2Δ lys2Δ/+ met15Δ/+ ura3Δ/ura3Δ prp43Δ/+*	ATCC # 4024487
JK203	*MATa/MATalpha his3Δ/Δ leu2Δ/Δ lys2? met15? ura3Δ/Δ PRP43Δ/Δ* pJK22	This study
JK207	*MATa/MATalpha his3Δ/Δ leu2Δ/Δ lys2? met15? ura3Δ/Δ PRP43Δ/Δ* pJK22 & pJK21- *PRP:* ddx-15	This study
JK208	*MATa/MATalpha his3Δ/Δ leu2Δ/Δ lys2? met15? ura3Δ/Δ PRP43Δ/Δ* pJK22 & pJK21- *TDH:* ddx-15	This study
JK205	*MATa/MATalpha his3Δ/Δ leu2Δ/Δ lys2? met15? ura3Δ/Δ PRP43Δ/Δ* pJK22 & pJK23	This study
